# The Accuracy of Conformation of a Generic Surface Mesh for the Analysis of Facial Soft Tissue Changes

**DOI:** 10.1371/journal.pone.0152381

**Published:** 2016-04-19

**Authors:** Man Yan Cheung, Anas Almukhtar, Andrew Keeling, Tai-Chiu Hsung, Xiangyang Ju, James McDonald, Ashraf Ayoub, Balvinder Singh Khambay

**Affiliations:** 1 Faculty of Dentistry, University of Hong Kong, Hong Kong, China; 2 School of Dentistry, University of Leeds, Leeds, United Kingdom; 3 Glasgow Dental School, University of Glasgow, Glasgow, United Kingdom; University of North Carolina at Chapel Hill, UNITED STATES

## Abstract

**Purpose:**

Three dimensional analysis of the face is required for the assessment of complex changes following surgery, pathological conditions and to monitor facial growth. The most suitable method may be “dense surface correspondence”.

**Materials and Methods:**

This method utilizes a generic facial mesh and “conformation process” to establish anatomical correspondences between two facial images. The aim of this study was to validate the use of conformed meshes to measure simulated maxillary and mandibular surgical movements. The “simulation” was performed by deforming the actual soft tissues of the participant during image acquisition. The study was conducted on 20 volunteers and used 77 facial landmarks pre-marked over six anatomical regions; left cheek, right cheek, left upper lip, philtrum, right upper lip and chin region. Each volunteer was imaged at rest and after performing 5 different simulated surgical procedures using 3D stereophotogrammetry. The simulated surgical movement was determined by measuring the Euclidean distances and the mean absolute x, y and z distances of the landmarks making up the six regions following digitization. A generic mesh was then conformed to each of the aligned six facial 3D images. The same six regions were selected on the aligned conformed simulated meshes and the surgical movement determined by determining the Euclidean distances and the mean absolute x, y and z distances of the mesh points making up the six regions were determined.

**Results:**

In all cases the mean Euclidian distance between the simulated movement and conformed region was less than 0.7mm. For the x, y and z directions the majority of differences in the mean absolute distances were less than 1.0mm except in the x-direction for the left and right cheek regions, which was above 2.0mm.

**Conclusions:**

This concludes that the conformation process has an acceptable level of accuracy and is a valid method of measuring facial change between two images i.e. pre- and post-surgery. The conformation accuracy is higher toward the center of the face than the peripheral regions.

## Introduction

Three-dimensional facial anthropometry has passed through many stages of development during the last few decades. Landmark based analysis was one of the earlier stages of facial anthropometry [1–3]. However, this method was criticised for its shortage in representing the soft tissue continuum by relying on only a few selected points, in addition to the questionable validity of landmarks based soft tissue analysis [4, 5]. Colour coded inter-surface distance (Hausdorff distance) maps were applied to analyse facial morphological changes. This method was frequently used for assessment of the variations of facial features in various populations [4–6] and for the evaluation of facial changes following specific surgical procedure [7, 8]. The method was based on calculation of the mean distance between the aligned surfaces. However, the lack of anatomical correspondence was one of the main shortcoming of the method [9, 10].

The use of generic meshes for the analysis of the geometry of biological structures has been previously suggested [11]. A generic facial mesh is a digitally constructed surface mesh that has the same shape as a typical human face. It consists of a known number of triangles and therefore a known number of points or vertices [12]. It is used to overcome the problem of two 3D surface meshes normally having broadly similar shapes but a different number of triangles; making it difficult to directly relate one point on one mesh to the same point on the other mesh. If the generic mesh is “wrapped” around two different 3D facial images, each new generic mesh will have the shape of each of the original 3D images and both new generic meshes will now have the same number of triangles and vertices. Since a point on one generic mesh is the same point on the other, direct anatomical correspondence can be achieved. The application of generic surface meshes allows comprehensive analysis using “dense correspondence analysis” of 3D human facial images using all the point making up the generic mesh providing a comprehensive quantitative evaluation of the examined surfaces.

In order to apply the generic mesh in constructing dense correspondence for facial analysis, the universal shape and geometry of the mesh were modified to resemble, more closely, the facial geometry and shape of each of the studied patient’s original 3D images. This was accomplished through a process known as “conformation” (elastic deformation) which creates a patient specific mesh. In the “conformed” mesh the number of vertices, triangular and vertices indices were preserved, the shape and geometry were specific to the studied case.

In the analysis of the effect of orthognathic surgery on facial morphology, the conformation process is applied to elastically deform the generic mesh to fit more specifically the 3D facial image before and after surgery. This process produces two meshes with the same number of vertices with identical indices so that each vertex represents a corresponding point on both pre- and post-operative conformed meshes.

The conformation process relies on an initial manual identification of corresponding “guide” landmarks on both surfaces to match certain corresponding anatomical features. This constitutes the basis for the final automated process of conformation [12]. The accuracy of the conformation process is a key element of a successful dense correspondence surface analysis. Previous studies reported on the high accuracy of the conformation process, this was based on surface distance analysis [11].

Therefore, the aim of this study was to validate the use of conformed meshes with anatomical correspondence to measure simulated maxillary and mandibular surgical movements. The “simulation” was performed by deforming the actual soft tissues of the participant during image acquisition. The null hypothesis was that there were no statistical differences in the Euclidian distances between the simulated movements of the chin, upper lip and the cheek regions and the conformed mesh (p<0.05). The second null hypothesis was that these differences, in the x, y and z directions were not equal to 1.0mm (p<0.05) as errors above these we thought to be clinically significant.

## Materials and Methods

**Fig 1:**
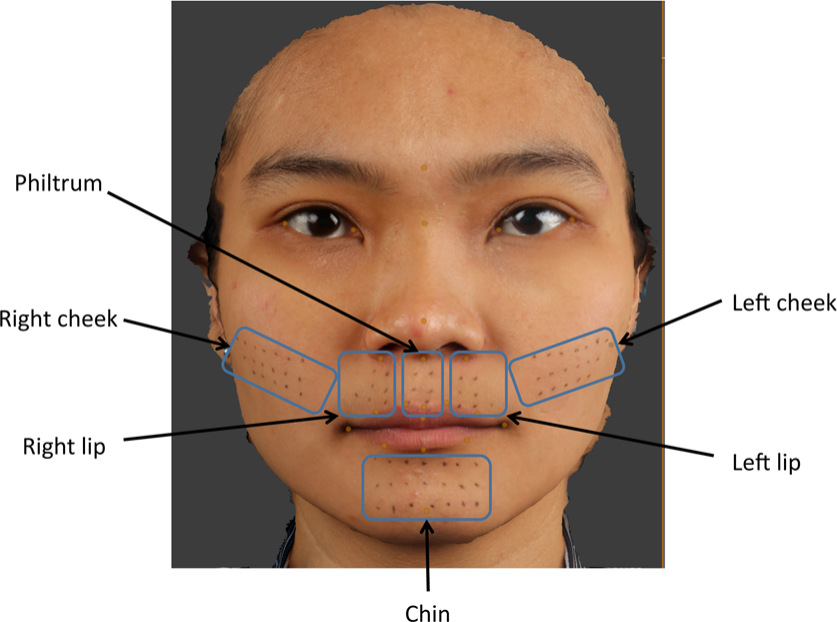
3D image showing 77 facial pre-marked landmarks; left cheek (18 landmarks), right cheek (18 landmark), left upper lip (7 landmarks), philtrum (6 landmarks), right upper lip (7 landmarks) and chin region (21 landmarks).

The individual in this manuscript has given written informed consent (as outlined in PLOS consent form) to publish their images. Following approval by the Institutional Review Board (IRB) of The University of Hong Kong and Hospital Authority Hong Kong West Cluster (UW 14–159); 20 individuals, 10 male and 10 female volunteers, were properly instructed and gave consent to participate in this study by signing the appropriate informed consent paperwork. For each individual 77 facial landmarks were pre-marked in six anatomical regions; left cheek (18 landmarks), right cheek (18 landmark), left upper lip (7 landmarks), philtrum (6 landmarks), right upper lip (7 landmarks) and chin region (21 landmarks), Fig 1. The number and anatomical distribution of the landmarks were standardized by constructing a template; made from hard-cured acrylic with 2mm diameter holes equally spaced 5mm apart. Each template was placed on each specific anatomical area and using a black non-permanent fine eyeliner pen (Dodo Japan Co., Ltd. Tokyo, Japan) the landmarks were placed on the patients skin, the template was removed prior to imaging. Male volunteers with facial hair were excluded in this study.

### Image acquisition

A 3D photograph was taken using the Di3D stereophotogrammetry system (Dimensional Imaging Ltd, Hillington, Glasgow, UK). Each volunteer was imaged in rest position as a baseline mesh. To assess simulated soft tissue mandibular surgical changes each volunteer was imaged with their mandible protruded, displaced to the left and displaced to the right. To assess simulated maxillary surgery and the associated upper lip changes additional images were taken with a dental cotton wool roll placed high up in the labial sulcus in the anterior maxillary region only and again with a cotton wool roll placed at the on the labial surfaces of the upper anterior teeth only. Prior to image capture patients were instructed on how to achieve the facial movement required for the study, guided by the operator. In total 6 images were produced for each volunteer, one baseline at rest, three assessing mandibular change and two maxillary change. All meshes were saved as Wavefront (.OBJ) file format for analysis.

### Mesh alignment

For each patient the baseline image (at rest) was imported into Di3DView software (Di3DView, Version 6.6) and regarded as the “target” mesh onto which each of the five remaining meshes were aligned one at a time. The alignment procedure, using ICP, involved selecting an area on the baseline mesh which remained unchanged between captures, i.e. forehead, left and right temporal region and nasal bridge. The alignment procedure then translated and rotated the simulated surgical mesh onto the baseline mesh to produce the “best-fit” based on the surface region. The number of iterations was set to 500 and using 20% of the vertices within the region. Each aligned mesh was saved in its new position in 3D space as an.OBJ file.

### Actual movement

**Fig 2:**
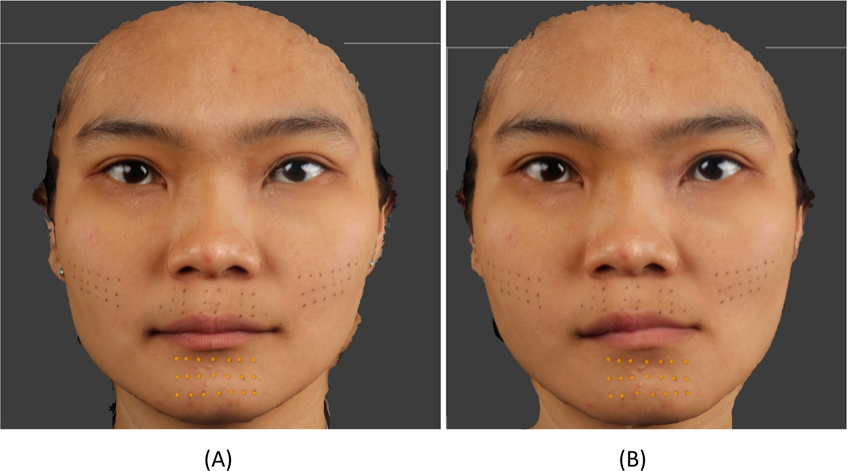
21 chin region landmarks used to measure surgical simulation of left mandibular displacement (B) from the baseline rest image (A).

Following alignment the Euclidian distances and the absolute distances in the x, y and z directions between corresponding landmarks on the baseline mesh and each of the simulated surgical meshes were used to assess the actual soft tissue change of each region. This was carried out by digitizing the landmarks in the region of interest using DiView. For example, to assess simulated mandibular advancement (mandible to left), following alignment of the baseline mesh with the mandibular to left mesh, the chin region landmarks were digitized on both meshes and exported, Fig 2. Using MATLAB (MathWorks, Cambridge, UK) the Euclidian distances between each of the 21 corresponding points together with the mean and standard deviation were calculated. The same procedure was carried out for the remaining simulated surgical movement. The chin region was used to assess the three different mandibular simulated surgical movement, whilst the left, right and philtrum regions of the upper lip and cheek regions were used to assess the maxillary simulated surgical movements.

### Conformed mesh generation

**Fig 3:**
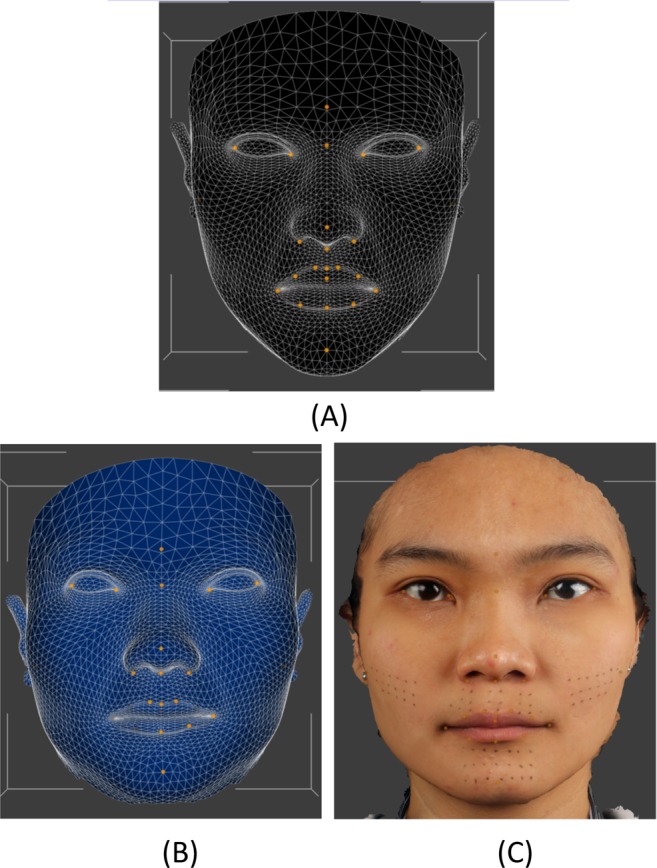
(A). Unconformed generic mesh. (B) Conformed mesh with similar shape of the simulated surgical mesh (C), but with the structure and topology.

A modified generic mesh provided by Dimensional Imaging was used for the conformation process. The generic mesh consisted of 3763 vertices and 7327 triangles. For each volunteer the generic mesh and baseline meshes were imported into DiView, twenty four landmarks (Table 1) were placed on the generic mesh and the same twenty four landmarks in same order were digitized on one of the baseline meshs. Using the “shape transfer” function in DiView the three dimensional positions of the twenty four corresponding landmarks on the generic mesh and simulated surgical mesh were used to calculate a warping function that moved all of the landmarks on the generic mesh to the exact position of the corresponding landmark on the simulated surgical mesh. This warping function was then applied to all of the vertices of the generic mesh so that it took on the approximate shape of the simulated surgical mesh. All of the vertices of the warped generic meshs were then projected along the surface normal onto the surface of the simulated surgical mesh, giving the generic mesh the exact shape of the simulated surgical mesh, but with the structured and known topology of the original generic mesh, Fig 3.

**Table 1:** Landmark definitions.

Glabella	The midpoint of the most prominent ridge between eye brows.
Nasion	Midpoint on the soft tissue contour of the base of the nasal root at the level of the frontonasal suture.
Exocanthion*	Soft tissue point located at the outer commissure of each eye fissure.
Endocanthion*	Soft tissue point located at the inner commissure of each eye fissure.
Alar base*	The base of the nostril.
Pronasale	The most protruded point of the apex nose identified in a lateral view of the rest position of the head.
Subnasale	Midpoint on the nasolabial soft tissue contour between the columella crest and the upper lip.
Cheilion*	Point located at each labial commissure.
Crista Philtre*	Point at each crossing of the vermillion line and the elevated margin of the philtrum.
Labrale Superius	Midpoint of the vermillion line of the upper lip.
Inferior Labrale Superius	A landmark on the upper lip located midway between Labrale Superius and Stomion Superius.
Labiale Inferius	Midpoint of the vermillion line of the lower lip.
Pogonion	Most anterior mid-point of the chin.
Midpoint between the crista philtre and Cheilion on the upper lip*	A landmark on the upper lip located midway between the Crista Philtre and Cheilion.
Midpoint Labiale Inferius and Cheilion on the lower lip*	A landmark on the lower lip located midway between Labiale Inferius and Cheilion.
Tragus*	A landmark on the midpoint of medial margin of the facial insertion of the tragus.

* bilateral left and right landmarks

### Region of interest selection

Having determined the actual movement of landmarks in the region of interest in the simulated surgical movements e.g. the chin region for mandibular movements, for comparison the same region needed to be located on the conformed mesh.

**Fig 4:**
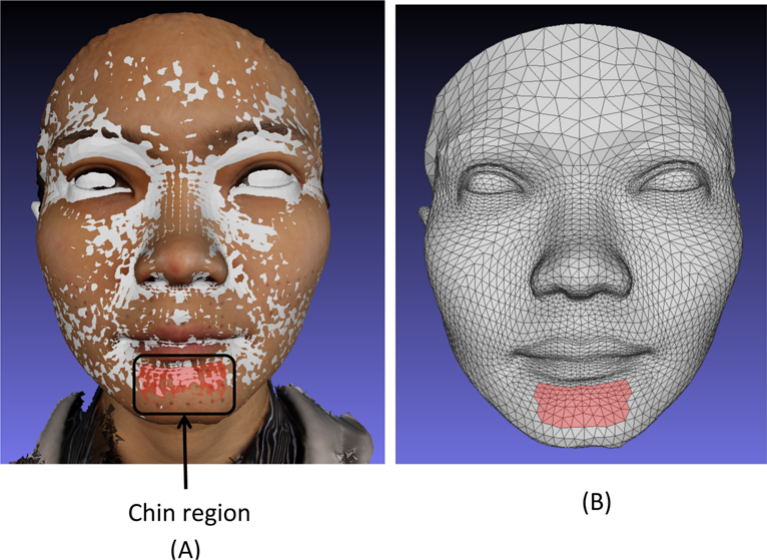
(A) Superimposed conformed mesh baseline (grey) and baseline textured image showing the 21 landmarks of the chin region and the region in red selected on the baseline conformed mesh. (B) The conformed mesh showing the chin region in red selected on the baseline conformed mesh used to assess simulated mandibular surgery. This allows direct comparison with the 21 chin region landmarks.

For each patient the original baseline 3D textured image and their baseline conformed mesh were imported into MeshLab software (STI-CNR, Rome, Italy; http://meshlab.sourceforge.net/). Using the “select face” function in MeshLab the triangles on the conformed mesh that represented the similar region of interest on the textured image was selected, Fig 4. This was repeated for each of the six regions of interest on the conformed baseline mesh. Each conformed baseline mesh image and selected region was re-saved in Polygon file format (.PLY) with “flags” attached to the selected triangles within the file structure. The remaining simulated surgical movement.OBJ files were imported into MeshLab and re-saved as.PLY to produce a common file format. Using MATLAB the vertices associated with the “flagged” triangles were identified. Since all the images were conformed using the same generic mesh these flagged triangles and vertices could be identified on all the meshes and would represent the same region between them all, hence providing direct correspondence. The mean Euclidian distances and the mean absolute distances in the x, y and z directions between corresponding vertices making up the region were calculated.

### Error study

To evaluate intra-operator error all the images of 10 volunteers chosen at random were selected and the 24 anatomical landmarks used in the conformation process were re-landmarked and used to re-conform all the images after four week interval. The Euclidian distances and the absolute distances in the x, y and z directions between the simulated surgical movements and the corresponding region movements between the first and second digitisations were used to analyse the error of the method. Systematic error was assessed using a paired *t* tests and random error assessed by coefficients of reliability.

## Results

### Measurement error

No systematic errors were observed. All coefficients of reliability were above 90%. All the differences in the x, y and z coordinates and the Euclidian distances between the repeated measurements showed no significant difference and were within ±0.4mm.

### Euclidian distances between the simulated surgical movement and the measurements based on the conformed mesh region

A two sample t-test showed no significant differences between the simulated surgical movement and the measurements based on the conformed mesh region (p<0.05). Overall, the mean Euclidian difference between the simulated movement and region ranged from 0.2mm (±0.2mm) to 0.7mm (± 0.5mm). The difference was smallest for the upper lip, left lip and philtrum region in the low advancement simulation and largest for the chin region in the left mandibular displacement simulations, Table 2.

**Table 2:** Euclidian distances between the simulated surgical movement and the measurements based on the conformed mesh region.

Simulated surgical movement	Region	Mean simulated movement (mm)	SD (mm)	Mean Euclidian distance between simulated movement and region (mm)	SD (mm)	95% CI for mean difference (mm)		p-value
						Upper	Lower	
Mandible to left	Chin	8.8	3.3	0.7	0.5	-2.3	1.9	0.861
Mandible to right	Chin	8.3	3.6	0.6	0.4	-1.8	1.3	0.832
Mandibular protrusion	Chin	7.3	1.8	0.3	0.3	-1.2	1.2	0.949
**Overall mandibular movement**	**Chin**	**-**	**-**	**0.5**	**0.5**	**-1.2**	**1.0**	**0.816**
High advancement	Upper lip	5.2	1.4	0.3	0.5	-0.8	1.1	0.728
High advancement	Right lip	5.0	1.2	0.4	0.3	-0.7	1.1	0.666
High advancement	Left lip	5.3	1.4	0.3	0.3	-0.9	1.0	0.876
High advancement	Philtrum	4.9	1.6	0.3	0.3	-1.2	1.1	0.971
Low advancement	Upper lip	5.2	1.9	0.2	0.2	-1.1	1.3	0.844
Low advancement	Right lip	5.1	1.9	0.4	0.3	-1.0	1.5	0.643
Low advancement	Left lip	5.5	1.6	0.2	0.2	-1.0	1.1	0.876
Low advancement	Philtrum	4.9	2.1	0.2	0.2	-1.3	1.5	0.875
**Overall advancement**	**Upper lip**	**-**	**-**	**0.3**	**0.3**	**-0.3**	**0.5**	**0.516**
High advancement	Cheek	2.5	1.2	0.6	0.5	-0.6	0.6	0.980
Low advancement	Cheek	2.4	1.5	0.5	0.6	-0.7	0.6	0.863
**Overall advancement**	**Cheek**	**-**	**-**	**0.6**	**0.5**	**-0.5**	**0.4**	**0.833**

### X, y and z distances between the simulated surgical movement and the measurements based on the conformed mesh region

**Fig 5:**
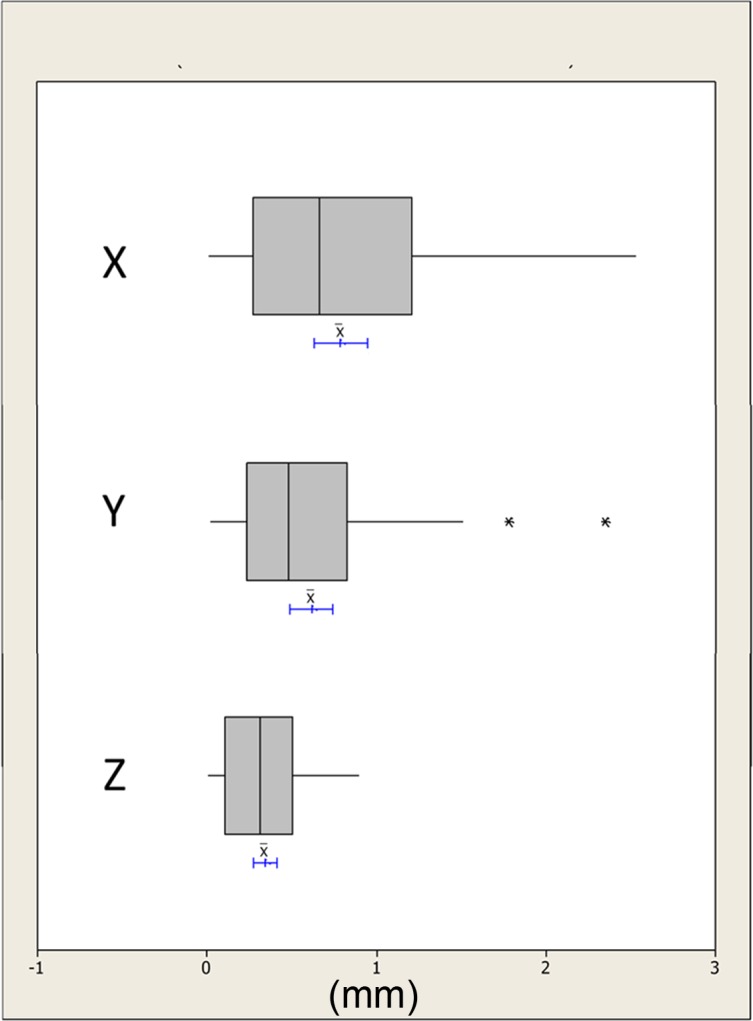
Boxplots showing the median, interquartile range (grey box) and outliers (*) for the x, y and z distances between all the simulated mandibular surgical movements i.e. left, right and protrusion and the measurements based on the conformed mesh chin region. The blue lines indicate the 95% confidence intervals for the mean differences.

**Fig 6:**
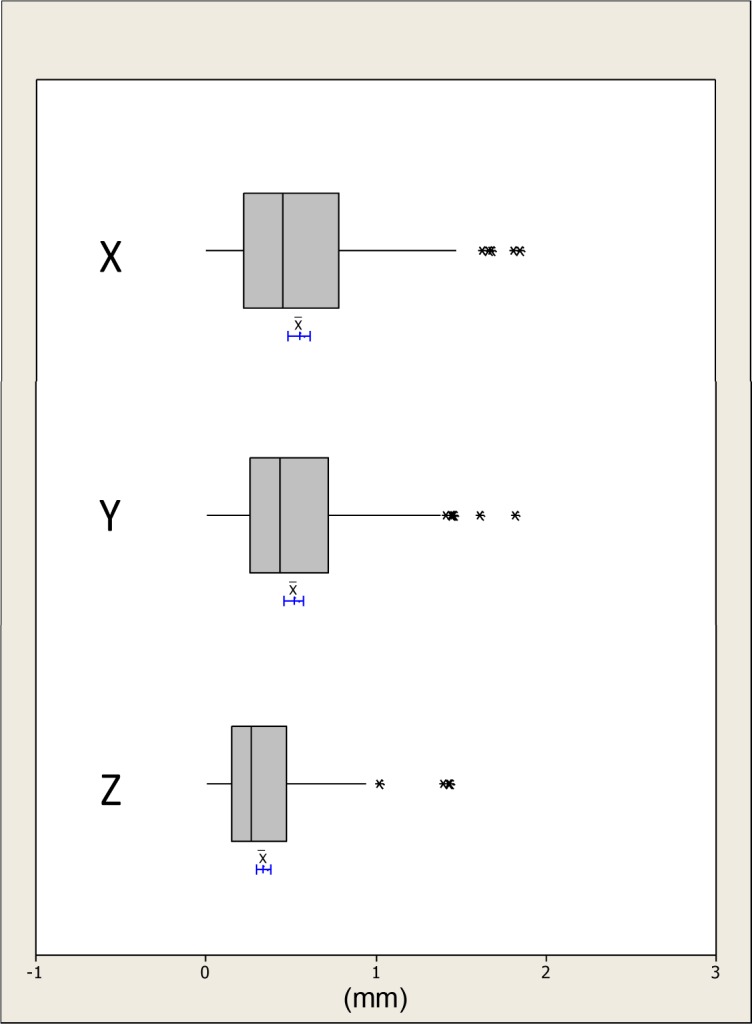
Boxplots showing the median, interquartile range (grey box) and outliers (*) for the x, y and z distances between all the simulated maxillary surgical movements i.e. high and low advancement and the measurements based on the conformed mesh upper lip, left lip, philtrum and right lip regions. The blue lines indicate the 95% confidence intervals for the mean differences.

**Fig 7:**
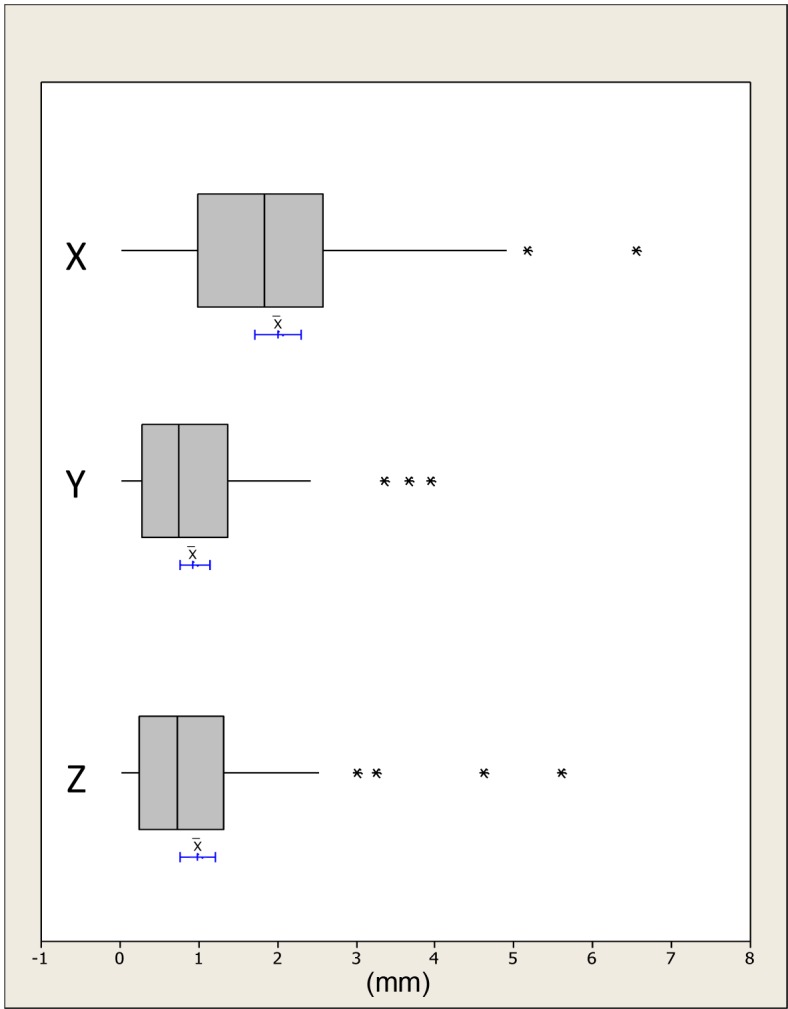
Boxplots showing the median, interquartile range (grey box) and outliers (*) for the x, y and z distances between all the simulated maxillary surgical movements i.e. high and low advancement and the measurements based on the conformed mesh cheek region. The blue lines indicate the 95% confidence intervals for the mean differences.

The absolute distances were plotted and checked for outliers; any human error was accounted for and no data points were rejected, Figs 5–7. The differences in the x, y and z mean absolute distances of the chin region between the simulated surgical mandibular movements compared to the corresponding movements determined using the conformed generic mesh ranged from 0.9mm (±0.7mm) for right simulated displacement in the x-direction to 0.3mm (±0.3mm) in the z-direction for left and protrusive mandibular movements. All the mean absolute differences were statistically significantly less than 1.0mm except in the x-direction for mandibular displacement to the left and right. This was confirmed by the 95% CI range with an upper limit of 1.1mm and 1.3mm for left and right displacement respectively.

The differences in the x, y and z absolute distances of the upper lip, right lip, left lip and philtrum regions between the simulated surgical movements compared to the corresponding movements determined using the conformed generic mesh were all statistically significantly less than 1.0mm except in the x-direction for the left lip region. This was confirmed with an upper 95% CI of 1.0mm, Table 3.

**Table 3:** Differences in the x, y and z mean absolute distances between the simulated surgical mandibular movements compared to the corresponding region on the conformed generic mesh.

Simulated surgical movement	Region and direction		Mean (mm)	SD (mm)	95% CI for difference (mm)		p-value
					Upper	Lower	
Mandible to left	**Chin**	**x**	0.8	0.6	1.1	0.6	**0.232**
		**y**	0.6	0.3	0.7	0.4	0.000
		**z**	0.3	0.3	0.5	0.2	0.000
Mandible to right	**Chin**	**x**	0.9	0.7	1.3	0.6	**0.717**
		**y**	0.6	0.5	0.3	0.1	0.001
		**z**	0.4	0.3	0.5	0.2	0.000
Mandibular protrusion	**Chin**	**x**	0.6	0.5	0.8	0.4	0.001
		**y**	0.7	0.6	1.0	0.4	0.034
		**z**	0.3	0.3	0.5	0.2	0.000
High advancement	**Upper lip**	**x**	0.5	0.4	0.7	0.3	0.000
		**y**	0.3	0.2	0.4	0.2	0.000
		**z**	0.2	0.2	0.3	0.2	0.000
	**Right lip**	**x**	0.7	0.6	1.0	0.4	0.026
		**y**	0.7	0.4	0.9	0.5	0.005
		**z**	0.4	0.3	0.5	0.2	0.000
	**Left lip**	**x**	0.8	0.5	1.0	0.6	**0.091**
		**y**	0.5	0.4	0.7	0.3	0.000
		**z**	0.4	0.2	0.5	0.3	0.000
	**Philtrum**	**x**	0.5	0.4	0.7	0.3	0.000
		**y**	0.4	0.3	0.6	0.3	0.000
		**z**	0.3	0.2	0.4	0.2	0.000
	**Left Cheek**	**x**	2.1	1.3	2.7	1.5	0.001
		**y**	1.1	1.0	1.6	0.7	**0.568**
		**z**	1.4	1.4	2.0	0.7	**0.252**
	**Right Cheek**	**x**	2.1	1.5	2.8	1.4	0.003
		**y**	1.0	1.1	1.5	0.5	**0.854**
		**z**	1.0	1.1	1.5	0.5	**0.884**
Low advancement	**Upper lip**	**x**	0.4	0.3	0.5	0.3	0.000
		**y**	0.4	0.3	0.5	0.3	0.000
		**z**	0.3	0.2	0.4	0.2	0.000
	**Right lip**	**x**	0.4	0.4	0.6	0.3	0.000
		**y**	0.7	0.4	0.9	0.5	0.002
		**z**	0.4	0.3	0.5	0.2	0.000
	**Left lip**	**x**	0.6	0.5	0.9	0.4	0.003
		**y**	0.6	0.4	0.8	0.4	0.000
		**z**	0.4	0.3	0.5	0.2	0.000
	**Philtrum**	**x**	0.4	0.3	0.5	0.3	0.000
		**y**	0.5	0.3	0.7	0.4	0.000
		**z**	0.4	0.3	0.5	0.2	0.000
	**Left Cheek**	**x**	1.9	1.2	2.5	1.4	0.003
		**y**	0.9	0.6	1.2	0.6	**0.399**
		**z**	0.9	0.6	1.3	0.6	**0.729**
	**Right Cheek**	**x**	1.9	1.3	2.5	1.3	0.007
		**y**	0.8	0.7	1.1	0.4	**0.158**
		**z**	0.7	0.7	1.0	0.3	**0.067**

## Discussion

Establishing an accurate conformation process (elastic deformation) of the generic facial mesh to resemble the detailed anatomy of the face is essential for a valid application of dense correspondence analysis to evaluate morphological changes. Stereophotogrammetry was the chosen facial capture method, in this study, to avoid exposing the volunteers to harmful radiation. At the same time, the method produced a three dimensional surface image of the face that contained the texture information which facilitated landmarks identification.

Previous studies on image conformation and elastic deformation have evaluated the accuracy of the process using the mean surface distance measurement [11, 12]. The deficiency of this approach lies in the fact that the distances were between the closest points of the two surface meshes, generic mesh and conformed mesh, not the actual correspondences. It is quite possible for the meshes to slide over each other during the conformation process, measuring the closest distance between two meshes would not detect this source of errors. Therefore, it was not possible to conduct this study on retrospective pre- and post-surgical data. It was necessary to compare the measurements obtained by the conformation process against simulated surgical movements in the maxilla and the mandible. The following movements were chosen, deviation of the mandible left and right representing correction of facial asymmetry and protrusive movements representing anterior-posterior changes. In addition simulated maxillary changes at a high level, immediately under the nose and low level or labial changes were also assessed.

It was not necessary to track every vertex on the conformed mesh to evaluate the accuracy of the process, only the regions of interest. The chin region for mandibular movements and the upper lip and cheek regions for the maxilla were chosen as being clinically relevant regions. Although this was not a comprehensive surface analysis, its robustness was maximized by selecting a set of landmarks that represented various anatomically relevant regions of the face. Two methods of measuring the disparities between the two surface meshes were considered, the absolute distances in the x, y and z directions and the Euclidean distance. Both of these measurements describe the magnitude of the error but not the direction. Using the arithmetic mean would produce artificially small results, as any positive and negative values would cancel each other out.

Ordinarily a comprehensive landmarking error study would have been performed but this would have been of limited value, as their effect on the conformation process would remain unknown. To address this problem the error of the whole procedural pipeline was conducted which indicated the differences in the x, y and z coordinates and the Euclidian distances between the repeated measurements showed no significant difference and were within ±0.4mm; which was thought to be clinically satisfactory. The use of pre-landmarking to determine the actual movement of the selected region during each simulated surgical movement significantly reduced the landmarking error [13].

The results from this study showed that using the proposed method of analysis and the three stages of mesh alignment, generic mesh conformation and region selection, the mean Euclidian distance between the baseline mesh and simulated surgical movement was not statistically different to the actual changes (p<0.05). For all mandibular movements the mean Euclidian distance difference for the chin region was 0.5mm (±0.5mm) (95% CI -1.2mm to 1.0mm), for upper lip region was 0.3mm (±0.3mm) (95% CI -0.3mm to 0.5mm) and for the cheek regions were 0.6mm (±0.5mm) (95% CI -0.5mm to 0.4mm). For the x, y and z directions the majority of differences in the mean absolute distances were less than 1.0mm except in the x-direction for the left and right cheek regions which were associated with an error of over 2.0mm and the chin region during right mandibular displacement (1.2mm). The upper lip region was associated with less error compared to the peripheral regions i.e. cheeks and chin. This might be due to the lower number of landmarks used during initial alignment during conformation and lack of distinguished surface topography upon which the elastic deformation relied. Given the lack of lateral anatomical landmarks in the region of the chin which could be used during the conformation process there will always probably be a higher magnitude of error.

This study has validated and determined the accuracy of measuring facial soft tissue changes in three dimensions using conformed generic meshes. It should now be possible to take a patients pre-operative and post-operative 3D facial scan, align them on stable structures i.e. the forehead, conform the generic meshes and determine the 3D regional soft tissue change following surgery. Based on the anatomical correspondence developed, this method of analysis produces a wealth of information that was previously unavailable. The limitations are that the changes lateral to the facial midline i.e. the cheeks regions are associated with a higher degree of inaccuracy which maybe clinically significant. It may be possible to improve this by using more landmarks during the conformation process but reproducible anatomical landmarks are sparse in this region i.e. cheek area. Based on a recently published study it will be possible to determine the 3D soft tissue changes of the soft tissue as a result of the underlying 3D hard tissue changes following orthognathic surgery using conebeam CT (CBCT) data [14]. This was however beyond the scope of the present study and taking CBCT of healthy volunteers to assess mandibular changes was unethical. The study will in the future be expanded to include orthognathic patients.

## Conclusions

The conformation process has an acceptable level of accuracy and is a valid method of measuring facial change between two images i.e. pre- and post-surgery. This has broad clinical applications including the analysis of facial analysis, evaluation of the impact of orthognathic surgery in changing facial morphology, and monitoring of facial growth. The conformation accuracy is higher toward the center of the face than the peripheral regions.
